# AI-driven evidence synthesis: data extraction of randomized controlled trials with large language models

**DOI:** 10.1097/JS9.0000000000002215

**Published:** 2025-02-04

**Authors:** Jiayi Liu, Honghao Lai, Weilong Zhao, Jiajie Huang, Danni Xia, Hui Liu, Xufei Luo, Bingyi Wang, Bei Pan, Liangying Hou, Yaolong Chen, Long Ge

**Affiliations:** a Department of Health Policy and Health Management, School of Public Health, Lanzhou University, Lanzhou, China; b Evidence-Based Social Science Research Center, School of Public Health, Lanzhou University, Lanzhou, China; c College of Nursing, Gansu University of Chinese Medicine, Lanzhou, China; d Evidence-Based Medicine Center, School of Basic Medical Sciences, Lanzhou University, Lanzhou, China; e Key Laboratory of Evidence-Based Medicine of Gansu Province, Lanzhou University, Lanzhou, China; f Research Unit of Evidence-Based Evaluation and Guidelines, Chinese Academy of Medical Sciences (2021RU017), School of Basic Medical Sciences, Lanzhou University, Lanzhou, China; g Department of Health Research Methods, Evidence, and Impact, McMaster University, Ontario, Canada; h WHO Collaborating Center for Guideline Implementation and Knowledge Translation, Lanzhou, China

**Keywords:** data extraction, evidence synthesis, large language models, randomized controlled trials

## Abstract

The advancement of large language models (LLMs) presents promising opportunities to enhance evidence synthesis efficiency, particularly in data extraction processes, yet existing prompts for data extraction remain limited, focusing primarily on commonly used items without accommodating diverse extraction needs. This research letter developed structured prompts for LLMs and evaluated their feasibility in extracting data from randomized controlled trials (RCTs). Using Claude (Claude-2) as the platform, we designed comprehensive structured prompts comprising 58 items across six Cochrane Handbook domains and tested them on 10 randomly selected RCTs from published Cochrane reviews. The results demonstrated high accuracy with an overall correct rate of 94.77% (95% CI: 93.66% to 95.73%), with domain-specific performance ranging from 77.97% to 100%. The extraction process proved efficient, requiring only 88 seconds per RCT. These findings substantiate the feasibility and potential value of LLMs in evidence synthesis when guided by structured prompts, marking a significant advancement in systematic review methodology.

Highlights

- Our study provided structured prompts to guide Claude in extracting data according to a form by Cochrane Handbook.
- Claude guided by structured prompts achieved an overall correct rate of 94.77% (95% confidence interval: 93.66% to 95.73%), with a mean time spent for one RCT of 88 seconds.
- Claude guided by structured prompts demonstrated commendable accuracy in data extraction in RCTs, indicating its potential application value in evidence synthesis.

Evidence synthesis, as an important method in evidence-based medicine, involves combining information from multiple studies investigating the same topic^[1]^. Currently, the rapidly growing number of primary studies poses significant challenges to data extraction, a key part of evidence synthesis^[2]^. While semi-automated tools have been developed to assist this process, they exhibit constraints in efficacy and application^[3,4]^.

With the development of advanced large language models (llms), claude (https://www.anthropic.com/) has demonstrated unprecedented potential as a data extraction assistant. Building on our previous work examining LLMs’ capability in RCT risk of bias assessment^[5]^, this study evaluates Claude’s accuracy and efficiency in extracting data from randomized controlled trials through structured prompts.

## Methods for development and validation of a standardized data extraction prompt

This survey study was conducted between 10 August 2023, and 30 October 2023. A multidisciplinary panel including experts in evidence-based medicine, methodology, and computer science led the study. The experts identified data extraction domains and items according to the Cochrane Handbook’s data collection form, covering “Methods”, “Participants”, “Baseline characteristics”, “Outcomes”, “Data and analysis” and “Others”^[6]^. The panel developed and refined structured prompts through iterative testing on three RCTs until achieving complete accuracy (Supplementary eAppendix 1, http://links.lww.com/JS9/D897).

**Figure 1.**
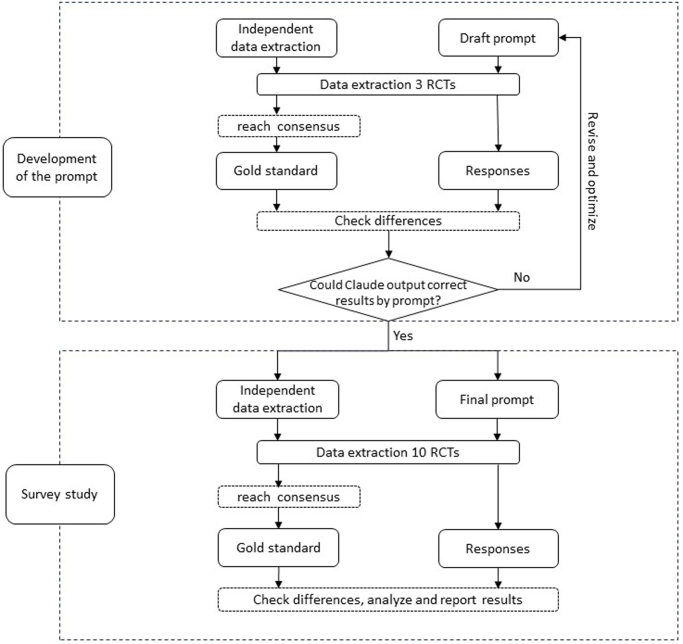
Flow diagram of the main study process.

We randomly selected ten RCTs from Cochrane reviews published between January 2023 and December 2023 as study samples. Claude was then used to extract data from these RCTs guided by the final prompts. The experts independently extracted data from the same RCTs to establish a gold standard through consensus. We assessed Claude’s performance by calculating the correct rate of extractions at overall, domain-specific, and item-specific levels, and recorded the time needed for each extraction to evaluate efficiency. The study workflow is illustrated in Fig. 1.

## Results of accuracy and efficiency assessment

Through iterative testing, we developed structured prompts for Claude’s data extraction, consisting of four components: instruction and role setting, general guidelines, data extraction guidelines, and output guidelines. Ten RCTs were randomly selected as our samples, covering various medical conditions including ophthalmic diseases, lung diseases, fractures, kidney disease, coronary artery disease, and neonatal care(Supplementary eAppendix 3, http://links.lww.com/JS9/D897).

### Accuracy

**Figure 2.**
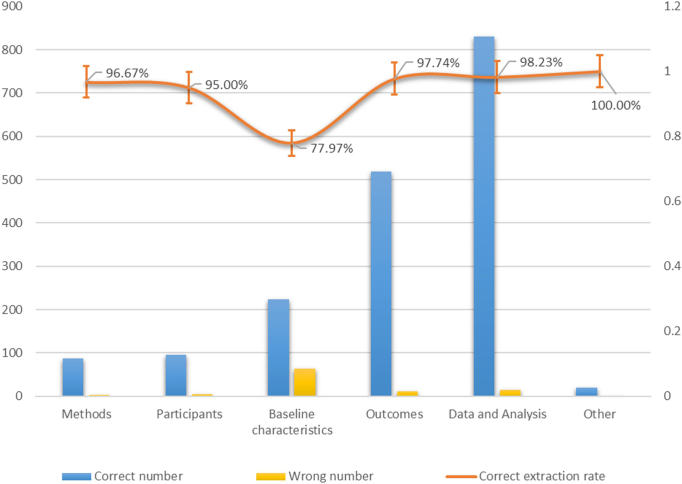
Domain-specific data extraction correct rates.

Claude achieved an overall correct rate of 94.77% (95% CI: 93.66% to 95.73%) in extracting 1873 items. At the domain-specific level, as shown in Fig. 2, the “Others” domain showed the highest correct rate of 100.00% (95% CI: 83.16% to 100.00%), while the “Baseline characteristics” domain showed the poorest performance with a correct rate of 77.97% (95% CI: 72.72% to 82.64%). The remaining domains, “Methods”, “Participants”, “Outcomes”, and “Data and analysis”, had correct rates exceeding 95% (range: 95.00% to 98.23%). At the item-specific level, as shown in Table 1, 51.72% (38/58) of all items achieved the correct extraction rate of 100%, and 20.68% (12/58) of them demonstrated a rate of more than 90%, while the remaining 13.79% (8/58) showed correct extraction rates under 90%. The main errors occurred in extracting participant numbers, particularly the item about number of participants excluded before randomization with the lowest correct rate at 9.09% (95% CI: 1.12% to 29.16%), and the item about number of participants assessed for eligibility with a correct rate of 18.18% (95% CI: 5.19% to 40.28%). The errors could be attributed to two main reasons: the data explicitly reported in the article was not recognized and the data not explicitly reported but could be inferred was not recognized(Supplementary eTable 3, http://links.lww.com/JS9/D897).

**Table 1:** The item-specific accuracy of data extraction by Claude

Item ID	Item	Total number	Correct number	Wrong number	Correct extraction rate	95% Confidence Interval	
						Lower limit	Upper limit
1.1	Study ID	10	10	0	100.00%		
1.2	Aim of study	10	10	0	100.00%		
1.3	Design	10	9	1	90.00%	55.50%	99.75%
1.4	Country	10	9	1	90.00%	55.50%	99.75%
1.5	Unit of allocation	10	10	0	100.00%		
1.6	Start date	10	10	0	100.00%		
1.7	End date	10	10	0	100.00%		
1.8	Duration of participation	10	9	1	90.00%	55.50%	99.75%
1.9	Ethical approval needed/obtained for study	10	10	0	100.00%		
2.1	Population description	10	10	0	100.00%		
2.2	Setting	10	7	3	70.00%	34.75%	93.33%
2.3	Inclusion criteria	10	10	0	100.00%		
2.4	Exclusion criteria	10	10	0	100.00%		
2.5	Method of recruitment of participants	10	10	0	100.00%		
2.6	Informed consent obtained	10	10	0	100.00%		
2.7	Total no. randomized	10	10	0	100.00%		
2.8	Clusters	10	10	0	100.00%		
2.9	Baseline imbalances	10	8	2	80.00%	44.39%	97.48%
2.10	Subgroups measured	10	10	0	100.00%		
3.1.1	Intervention name	22	22	0	100.00%		
3.1.2	Assessed for eligibility	22	4	18	18.18%	5.19%	40.28%
3.1.3	Excluded	22	2	20	9.09%	1.12%	29.16%
3.1.4	Randomized	22	22	0	100.00%		
3.1.5	Lost to follow-up	22	13	9	59.09%	36.35%	79.29%
3.1.6	Analyzed	22	18	4	81.82%	59.72%	94.81%
3.1.7	Excluded from analysis	22	11	11	50.00%	28.22%	71.78%
3.1.8	Age	22	21	1	95.45%	77.16%	99.88%
3.1.9	Female	22	22	0	100.00%		
3.1.10	Race/Ethnicity	22	22	0	100.00%		
3.1.11	BMI	22	22	0	100.00%		
3.1.12	Severity of illness	22	22	0	100.00%		
3.1.13	Co-morbidities	22	22	0	100.00%		
4.1.1	Outcome name	59	57	2	96.61%	88.29%	99.59%
4.1.2	Outcome definition	59	57	2	96.61%	88.29%	99.59%
4.1.3	Outcome measurement tool	59	57	2	96.61%	88.29%	99.59%
4.1.4	Unit of measurement	59	57	2	96.61%	88.29%	99.59%
4.1.5	Range of measurement	59	59	0	100.00%		
4.1.6	Is outcome/tool validated	59	57	2	96.61%	88.29%	99.59%
4.1.7	Evaluation type	59	59	0	100.00%		
4.1.8	Power	59	59	0	100.00%		
4.1.9	Analysis Method	59	57	2	96.61%	88.29%	99.59%
5.1.1	Outcome name	58	58	0	100.00%		
5.1.2	Intervention and comparison	58	58	0	100.00%		
5.1.3	Outcome type	55	55	0	100.00%		
5.1.4	Effect	58	58	0	100.00%		
5.1.5	LCI (Lower Confidence Interval)	55	55	0	100.00%		
5.1.6	UCI (Upper Confidence Interval)	55	54	1	98.18%	90.28%	99.95%
5.1.7	Discrete trend	56	56	0	100.00%		
5.1.8	No. with event in IG (Intervention Group)	57	57	0	100.00%		
5.1.9	Total in group in IG	57	57	0	100.00%		
5.1.10	No. with event in CG (Control Group)	57	57	0	100.00%		
5.1.11	Total in group in CG	57	57	0	100.00%		
5.1.12	Missing participants	56	47	9	83.93%	71.67%	92.38%
5.1.13	Participants moved from another group	56	51	5	91.07%	80.38%	97.04%
5.1.14	Time points measured	56	56	0	100.00%		
5.1.15	Subgroup	55	55	0	100.00%		
6.1	Study funding sources	10	10	0	100.00%		
6.2	Possible conflicts of interest	10	10	0	100.00%		

### Efficiency

Overall, Claude took an average of 88.80 seconds to extract data from one RCT, with approximately two correct items extracted per second. The extraction time varied according to the complexity of RCTs, particularly the number of groups and reported outcomes.

## The opportunities and challenges of LLMs in data extraction

In this survey study, we developed and validated structured prompts to guide Claude in extracting data from RCTs. Claude showed an overall high level of accuracy and efficiency, indicating its feasibility in data extraction. Although the “Baseline characteristics” domain showed the lowest correct rate, possibly due to the flexibility of article reporting, the errors were notably localized and patterned. Among 98 wrong extractions in 1873 items, 79 (80.61%) occurred in items about participant numbers, with most errors stemming from failure to infer implicit data (62.24%) or recognize explicit data (37.76%). This pattern suggests researchers could swiftly identify and rectify discrepancies through targeted review.

Compared to manual data extraction which typically takes 15-20 minutes per article, Claude significantly reduced the time to 88.80 seconds per RCT. While previous semi-automated tools have shown limited acceptance due to reliability and user-friendliness issues, Claude offers advantages in processing extensive text data, accessibility, and convenience. Through iterative attempts, we found that structured prompts with detailed domain and item explanations, rather than pre-formulated forms, were crucial for effective extraction.

As the study assessing LLM’s performance in data extraction, our research provides important evidence for improving evidence synthesis efficiency. Future studies with larger samples and different languages are needed to further validate these findings. Additionally, the development of reporting guidelines for LLM use in medical research, such as our proposed CHEER guidance, will be valuable for ensuring transparency^[7]^.

## Conclusion

In this survey study of the application of LLM in extracting data of RCT, we developed structured prompts and found that Claude was able to extract data efficiently and accurately, showing the application feasibility and value of LLM in systematic review production. By analyzing the errors that occurred, we found that many extraction errors were regular, which suggested that researchers could quickly find and correct errors.

## Data Availability

Data are not from the database; all data are presented in the supplementary material.
